# Professor David W. Denning an Extraordinary High Achiever in Clinical and Translational Science

**Published:** 2018-09

**Authors:** Vahid Khalaj

**Affiliations:** Medical Biotechnology Department, Biotechnology Research Center, Pasteur Institute of Iran, Tehran, Iran


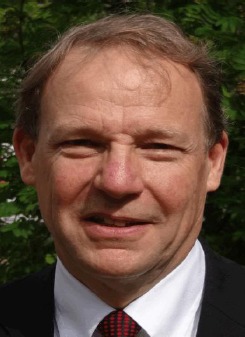


It is often wondered as to what part of all the research performed around the globe actually leaves the lab and reaches the real people and betters their lives. In case of Professor David W. Denning’s research, a large part does. As a prominent international face in the field of medical mycology, he translates his research into clinical applications and takes medical problems back to his lab, in order to develop potential solutions.

He is a professor of Infectious Diseases in Global Health and a consultant in infectious diseases and medical mycology, Wythenshawe Hospital and University of Manchester. He is also the founding president of the Global Action Fund for Fungal Infections (GAFFI) and the leader of Leading International Fungal Education (LIFE). He supervises a strong research group funded by grants from the UK, USA, and Europe, as well as multiple pharmaceutical companies, with research grant income of more than 22 million pounds to Manchester University, since 1990.

David Denning graduated from Guy’s Hospital in London in 1980 and was trained in London and Glasgow in internal medicine and infectious diseases, gaining additional experience in pediatrics, hematology, gastroenterology, and immunology. He, then, undertook research training at the MRC Clinical Research Centre, Harrow, UK (1985-87), followed by a three-year fellowship in diagnostic microbiology and infectious diseases with Professor David Stevens at Stanford University, Santa Clara Valley Medical Center (1987-1990). He was granted a personal chair by the Manchester University in 2005. He coordinated the international consortium sequencing the *Aspergillus fumigatus* genome (funded by NIH, Welcome Trust, and the Spanish government, 1997-2005). He is the Director of UK’s National Aspergillosis Centre, the world’s only such center based at Wythenshawe Hospital in Manchester. He has a continuing interest in the treatment of invasive aspergillosis and has been closely involved in the conduct and analysis of clinical trials of itraconazole, voriconazole, Amphotec (Amphocil), caspofungin, and micafungin. He maintains an active research program in many areas of antifungal therapy, genomics of Aspergillus, and antifungal resistance and has significant experience in both the preclinical and clinical evaluations of novel agents. He also works as a practicing physician at Wythenshawe Hospital with expertise in fungal infections, particularly aspergillosis and antifungal resistance, mainly in the immunocompromised patients.

Professor David Denning is the founder and the editor of the Aspergillus Website (with over 1 million pages read per month). His brilliant multifaceted contributions has entered him into several “Who’s Who” listings, including in the world (since 1996/7), in healthcare (since 1997), and in science and technology (since 2005). As a prominent internationally recognized scientist, he is asked to chair several international conferences, around the globe, including but not limited to “Advances against Aspergillosis”, “Trends in Invasive Fungal Infection”, and “Aspergillus Genetics and Genomes Focus Meeting”.

Professor Denning’s main clinical interests include fungal diseases, immunocompromised patients, and complex hospital infection problems. As a translational researcher, his research interests thus focus on antifungal susceptibility testing and resistance, pathogenesis of invasive aspergillosis, clinical studies of antifungal agents, *Aspergillus* genomics, and the burden of fungal infections globally and in different countries.

Unlike most researchers who remain confined to the boundaries of their research laboratories, David Denning has founded and promoted a number of successful businesses, making the newly developed drugs accessible to the market. In 1998, he originally founded the F2G Ltd. (antifungal drug discovery and development), and continues to serve as a clinical advisor. Later on, in 2006 he founded Myconostica Ltd. (molecular diagnostic tests for fungi) and served as its chief medical officer until 2011, when it was acquired by Lab21. He has also served and/or continues to serve as an expert consultant to multiple pharmaceutical companies with regard to antifungal drug discovery, including Merck, Pfizer, Schering-Plough, Astellas Pharma, SCYNEXIS, Cidara Theraputics, Biosergen, Pulmatrix, and Pulmocide.

As a pioneer in his field, he remains a long-standing member of the Infectious Diseases Society of America Aspergillosis Guidelines Group, the European Society for Clinical Microbiology and Infectious Diseases Aspergillosis Guidelines Group, and the British Society for Medical Mycology Standards of Care Committee. In October of 2017, he received the prestigious award of Eduard Drouhet Medal from the European Confederation of Medical Mycology (ECMM).

Professor David Denning’s outstanding publications mount to more than 500 papers, books, book chapters and lectures worldwide, which have attracted over 50,000 citations. Some of these publications have reported the results of his successfully lead major international collaborative projects in highly distinguished journals such as Nature, the New England Journal of Medicine, and the Lancet.

**Selected recent publications:**

- Denning DW, Bromley MJ. **How to bolster a sparse antifungal pipeline**. Science 2015; 347:1414-6.
- Denning DW, Cadranel J, Beigelman-Aubry C, Ader, F, Chakrabarti A, Blot S, Ullman A, Dimopoulos G, Lange C, European Society for Clinical Microbiology and Infectious Diseases and European Respiratory Society. **Chronic pulmonary aspergillosis–Rationale and clinical guidelines for diagnosis and management**. Eur Resp J 2016; 47: 45-68.
- Denning DW. **How the UNAIDS target of reducing annual AIDS deaths below 500,000 by 2020 can be achieved**. Phil Trans Roy Soc B 2017; 371: 20150468.

